# Protective Effect of Resveratrol on Kidney Disease and Hypertension Against Microplastics Exposure in Male Juvenile Rats

**DOI:** 10.3390/antiox13121457

**Published:** 2024-11-27

**Authors:** You-Lin Tain, Guo-Ping Chang-Chien, Shu-Fen Lin, Chih-Yao Hou, Chien-Ning Hsu

**Affiliations:** 1 Department of Pediatrics, Kaohsiung Chang Gung Memorial Hospital, Kaohsiung 833, Taiwan; tainyl@cgmh.org.tw; 2 College of Medicine, Chang Gung University, Taoyuan 330, Taiwan; 3 Institute of Environmental Toxin and Emerging-Contaminant, Cheng Shiu University, Kaohsiung 833301, Taiwan; guoping@csu.edu.tw (G.-P.C.-C.); linsufan2003@csu.edu.tw (S.-F.L.); 4 Super Micro Mass Research and Technology Center, Cheng Shiu University, Kaohsiung 833, Taiwan; 5 Center for Environmental Toxin and Emerging-Contaminant Research, Cheng Shiu University, Kaohsiung 833, Taiwan; 6 Department of Seafood Science, National Kaohsiung University of Science and Technology, Kaohsiung 811, Taiwan; chihyaohou@webmail.nkmu.edu.tw; 7 Department of Pharmacy, Kaohsiung Chang Gung Memorial Hospital, Kaohsiung 833, Taiwan; 8 School of Pharmacy, Kaohsiung Medical University, Kaohsiung 807, Taiwan

**Keywords:** hypertension, kidney disease, microplastics, resveratrol, oxidative stress, renin-angiotensin system, gut microbiota, short chain fatty acids

## Abstract

Global pollution stems from the degradation of plastic waste, leading to the generation of microplastics (MPs). While environmental pollutants increase the risk of developing hypertension and kidney disease, the effects of MP exposure on these conditions in children remain unclear. Resveratrol, a phenolic compound known for its antihypertensive and renoprotective properties, has gained attention as a potential nutraceutical. This study investigates the effects of resveratrol on kidney disease and hypertension induced by MP exposure in a juvenile rat model. Three-week-old male Sprague–-Dawley (SD) rats were randomly allocated into four groups (n = 8 per group): a control group, a low-dose MP group (1 mg/L), a high-dose MP group (10 mg/L), and a high-dose MP group receiving resveratrol (50 mg/L). By 9 weeks of age, MP exposure resulted in elevated blood pressure and increased creatinine levels, both of which were mitigated by resveratrol treatment. The hypertension and kidney damage induced by high-dose MP exposure were linked to oxidative stress, which resveratrol effectively prevented. Additionally, resveratrol’s protective effects against hypertension and kidney damage were associated with increased acetic acid levels, reduced renal expression of Olfr78, and decreased expression of various components of the renin-angiotensin system (RAS). Low- and high-dose MP exposure, as well as resveratrol treatment, differentially influence gut microbiota composition. Our findings suggest that targeting oxidative stress, gut microbiota, and the RAS through resveratrol holds therapeutic potential for preventing kidney disease and hypertension associated with MP exposure. However, further research is needed to translate these results into clinical applications.

## 1. Introduction

Plastic pollution is widely acknowledged as a critical global environmental issue [1]. The degradation of plastic waste results in microplastics (MPs) and nanoplastics (<5 mm and <1 mm, respectively), which can accumulate in the environment and potentially adversely impact human health [2]. The toxic effects of MPs are associated with their capacity to absorb and release harmful chemical pollutants. These MPs are increasingly found in various foods—such as drinking water, milk, and seafood—allowing them to enter our bodies through the food chain in unpredictable ways [2].

Hypertension and kidney disease are prevalent conditions with a complex bidirectional relationship [3,4]. While these conditions are most prevalent among adults, they may begin in childhood and have origins in fetal life [5]. Research has shown that exposure to environmental pollutants raises the risk of developing both hypertension and kidney disease in both adults and children [6]. However, the impact of MP exposure on these conditions, particularly in children, remain largely unexamined [6].

MPs can penetrate the gut epithelium and translocate to various organ systems, including the kidneys. The accumulation of MPs in kidneys has been observed in a few animal studies and a single human report [7,8]. However, the potential consequences of MP exposure on kidney function, the specific mechanisms involved, and their overall contribution to kidney disease and hypertension still require further investigation [9]. Several mechanisms have been proposed to explain environmental pollutant-induced kidney disease [6]. Key areas of investigation include oxidative stress, dysbiosis of the gut microbiota, and abnormal activation of the renin-angiotensin system (RAS).

Resveratrol is a phenolic compound known for its diverse biological functions and is widely used as a nutraceutical [10]. Growing evidence supports the antihypertensive and renoprotective effects of resveratrol [11]. Resveratrol is well known for its protective effects as an antioxidant [12]. Additionally, resveratrol possesses prebiotic properties that can alter gut microbiota [13]. Moreover, we previously found that resveratrol supplementation can protect adult progeny against hypertension induced by high-fat diet, which was linked to the restoration of the disrupted RAS [14]. These observations support that resveratrol might counteract major mechanisms behind pollutant-related kidney disease.

Despite the benefits of resveratrol mentioned above, only one study has investigated its protective effects against nanoparticle-induced endothelial dysfunction [15]. Given resveratrol’s role in mitigating hypertension and kidney disease induced by environmental pollutants, we aim to examine whether MP exposure induces hypertension and kidney injury in juvenile rats. Additionally, we evaluate the protective effects of resveratrol, focusing on gut microbiota, oxidative stress, and the RAS.

## 2. Materials and Methods

### 2.1. Animal Protocol

The Institutional Animal Ethics Committee of Kaohsiung Chang Gung Memorial Hospital approved this study (permit number: 2023081103, approval date: 28 September 2023). Sprague–Dawley (SD) rats were bred in an animal facility accredited by AAALAC International. Pregnant rats had unrestricted access to chow and water until delivery, and offspring were raised with their dams during the lactation period. After weaning, three-week-old male SD rats were randomly allocated into four groups (n = 8 per group): a control group receiving distilled water (CN), a low-dose group receiving 5 μM MP (1 mg/L; MPL), a high-dose group receiving 10 mg/L MP (MPH), and a high-dose group receiving MP with resveratrol (50 mg/L; MPHR). The negatively charged sulfate-modified polystyrene MPs were custom-designed and purchased from Magsphere Inc. (Pasadena, CA, USA; Lot Number: PSG5638). These polystyrene MPs are provided as a 50 mL solution (10% solids) with a particle size of 5.0 µm and a green color. The average particle diameter tolerance was within 10%. The dosages of MPs and resveratrol were based on previous studies involving rats [16,17]. Only male juvenile rats were utilized due to their higher susceptibility to developing hypertension earlier than females [18].

Noninvasive blood pressure (BP) measurements were conducted on conscious, trained rats using the tail-cuff method (CODA, Kent Scientific Corp., Torrington, CT, USA). The rats were acclimatized to the restraint holders for one week prior to the recording sessions. Each rat was euthanized at nine weeks of age. Prior to euthanasia, fecal samples were collected and stored at −80 °C until extraction. Both kidneys were excised and weighed. Plasma samples were collected, aliquoted, and stored at −80 °C. Creatinine concentrations in plasma were analyzed using an HP series 1100 high-performance liquid chromatography (HPLC) system (Agilent Technologies, Wilmington, DE, USA).

### 2.2. Determination of Plasma SCFAs

We analyzed SCFA concentrations in the plasma using a gas chromatograph–mass spectrometer (Agilent Technologies), following the methods described previously [19]. The three major SCFAs—acetic acid, propionic acid, and butyric acid—were quantified. Additionally, other SCFAs, such as isobutyric acid, isovaleric acid, and valeric acid, which are present in smaller amounts, were also measured. These SCFAs were separated using a DB-FFAP column, with 2-ethylbutyric acid serving as the internal standard.

### 2.3. Quantitative PCR

Total RNA was extracted from kidney cortex samples, followed by real-time quantitative PCR (qPCR), as previously detailed in our methods [19]. We analyzed the expression of SCFA-sensing G protein-coupled receptors (GPCRs), containing GPR41, GPR43, GPR109A, and olfactory receptor 78 (OlfR78). Additionally, we examined elements of the RAS, specifically angiotensin-converting enzyme 1 (ACE1), angiotensin II type 1 receptor (AT1R), renin, and the (pro)renin receptor (PRR). All samples were analyzed in duplicate, with 18S ribosomal RNA (18S rRNA) used as the reference gene for normalizing the qPCR data. Relative gene expression was determined using the comparative threshold cycle (Ct) method, with fold changes calculated using the formula 2^−ΔΔCt^ to compare target genes to the control. We included the primer sequences in Table 1.

### 2.4. Gut Microbiota Metagenomics

Microbial DNA was extracted from rat feces, and metagenomic analysis was performed using 16S ribosomal RNA genes [19]. Full-length 16S rRNA genes were amplified for PacBio sequencing using barcode primers that were designed for SMRTbell library preparation (Menlo Park, CA, USA). We used the QIIME2 software package for downstream analyses of the sequences [20]. From the amplicon sequence variants (ASVs) sequences, a phylogenetic tree was constructed using FastTree, and each cluster is taxonomically assigned. For α-diversity analysis, we employed the observed features and Shannon index, while β-diversity was assessed using analysis of similarities (ANOSIM) in conjunction with partial least squares discriminant analysis (PLSDA). Additionally, linear discriminant analysis (LDA) effect size was utilized to detect bacterial taxa with meaningfully different abundances between groups [21].

### 2.5. Immunohistochemistry for 8OHdG Staining

Since 8-hydroxydeoxyguanosine (8-OHdG) is a marker of oxidative DNA damage, we performed immunohistochemistry using the HRP-DAB detection method (BIOTnA Biotech, Kaohsiung, Taiwan), as previously described [19]. We incubated the primary antibody (8-OHdG 1:100, JaICA, Shizuoka, Japan) with the kidney sections. Immunostaining positive cells were quantified in 10 random sections in the renal cortex using Ventana Image Viewer version 3.2.0.

### 2.6. Statistical Analysis

Data are expressed as mean ± standard error of means (SEM). The differences between groups were analyzed by Tukey post hoc test of ANOVA. Results were considered statistically significant when the *p* value was less than 0.05. Analysis was completed using SPSS V17.0 software.

## 3. Results

### 3.1. Anthropometrics and Blood Pressure

**Figure 1:**
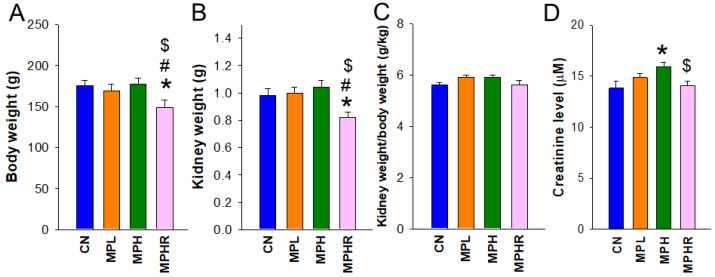

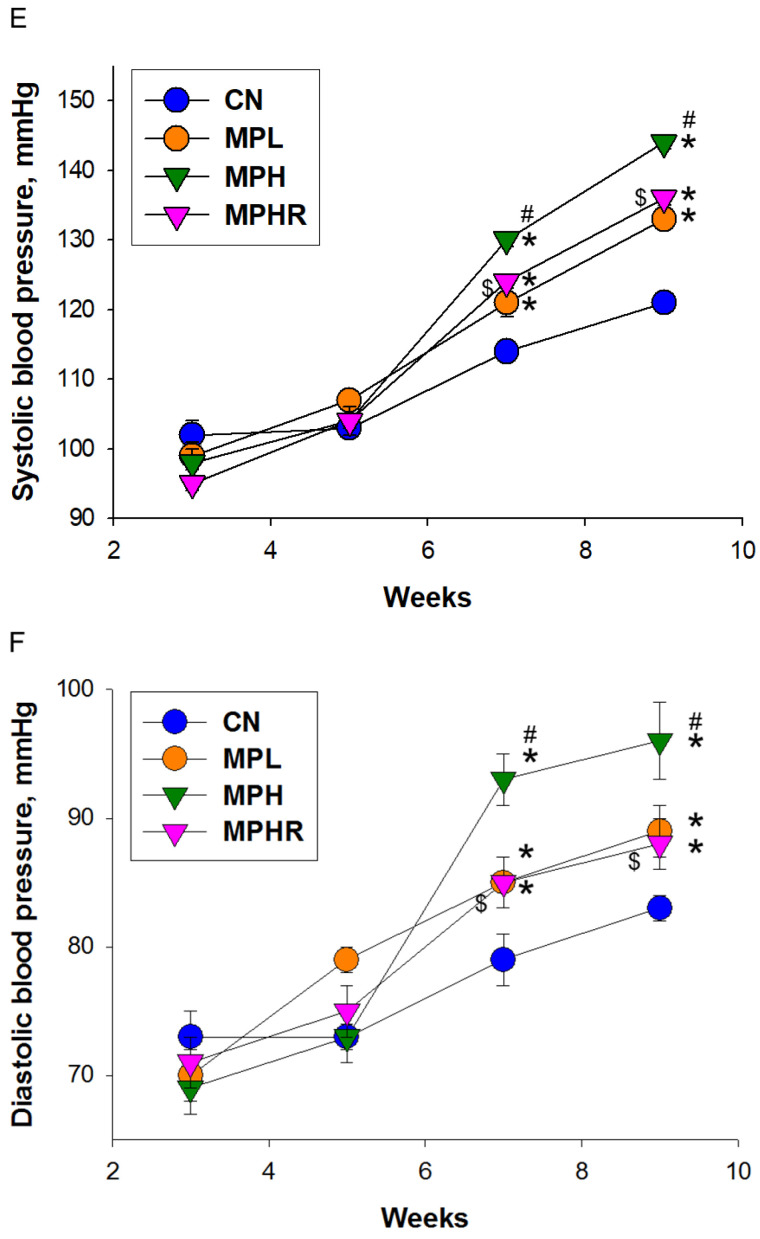
(**A**) Body weight, (**B**) kidney weight, (**C**) kidney weight-to-body weight ratio, and (**D**) plasma creatinine level. (**E**) Systolic blood pressure and (**F**) diastolic blood pressure at age 3 to 9 weeks. N = 8/group; * *p* < 0.05 vs. CN, # *p* < 0.05 vs. MPL; $ *p* < 0.05 vs. MPH. CN, control rats; MPL, rats received low-dose microplastics; MPH, rats received high-dose microplastics; MPHR, rats received high-dose microplastics and resveratrol.

MP exposure did not lead to rat mortality at nine weeks of age. Among the four groups, the MPHR group exhibited the lowest body weight (BW) and kidney weight (KW) (Figure 1A,B), although the KW-to-BW ratio remained comparable (Figure 1C). Figure 1D shows that high-dose MP exposure significantly elevated plasma creatinine levels (CN vs. MPH: 13.8 ± 0.7 μM vs. 15.9 ± 0.4 μM, *p* = 0.019). However, resveratrol supplementation restored these increases (MPHR vs. MPH: 14.1 ± 0.4 μM vs. 15.9 ± 0.4 μM, *p* = 0.011). Additionally, Figure 1E shows that low-dose MP exposure increased systolic blood pressure (BP) from eight to 12 weeks, with further elevation observed at high doses. Notably, the increases in systolic BP due to high-dose MP exposure were mitigated by resveratrol treatment. Similar to systolic BP, diastolic BP exhibited the same pattern (Figure 1F).

### 3.2. Plasma SCFAs and SCFA Receptors

Table 2 shows how MP and resveratrol affects plasma SCFAs concentrations. Low-dose MP exposure resulted in reduced plasma levels of acetic acid in the MPL group compared to the CN group. In contrast, resveratrol significantly increased plasma levels of acetic acid compared to both the MPL and MPH groups. Additionally, MPH rats exhibited lower plasma concentrations of isovaleric acid compared to the CN group. We found plasma concentrations of propionic acid, isobutyric acid, butyric acid, or valeric acid did not differ among the four groups.

**Figure 2:**
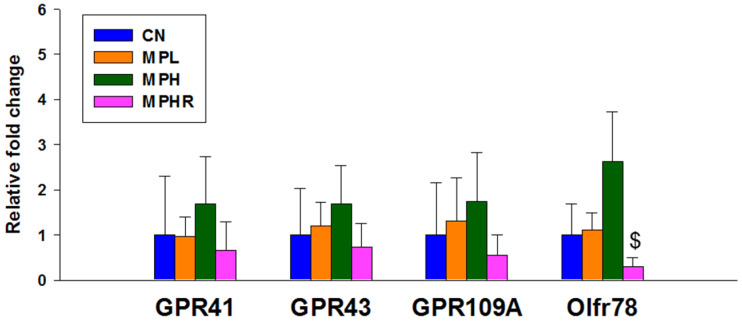
Renal mRNA expression of SCFA receptors at 9 weeks. This study investigates the mRNA expression levels of G protein-coupled receptors (GPCRs) GPR41, GPR43, GPR109A, and olfactory receptor 78 (OlfR78) in the rat kidneys. N = 8/group. $ *p* < 0.05 vs. MPH. CN, control rats; MPL, rats received low-dose microplastics; MPH, rats received high-dose microplastics; MPHR, rats received high-dose microplastics and resveratrol.

Figure 2 illustrates that resveratrol significantly reduced renal Olfr78 expression in the MPHR group compared to the MPH group. Although high-dose MP exposure appeared to increase their expression, this change did not reach statistical significance. No significant differences were found in the expression of GPR41, GPR43, and GPR109A among the four groups.

### 3.3. Gut Microbiota Composition

**Figure 3:**
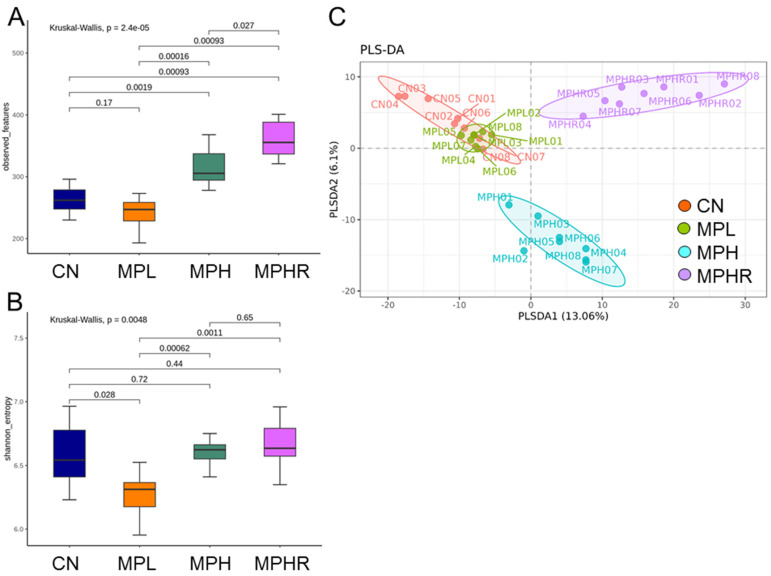
α-diversity among groups: (**A**) observed feature and (**B**) Shannon index. β-diversity among groups: (**C**) partial least squares discriminant analysis (PLSDA). Each spot represents the microbiota of a specific sample, with the color indicating the group to which the sample belongs. N = 8/group. CN, control rats; MPL, rats received low-dose microplastics; MPH, rats received high-dose microplastics; MPHR, rats received high-dose microplastics and resveratrol.

We analyzed two α-diversity metrics, observed features and Shannon index, to account for microbiome richness and evenness. Our results indicated statistically significant differences in α-diversity among the four groups, with observed features (*p* = 2.4 × 10^−5^) and Shannon index (*p* = 0.0048). The MPHR group exhibited the highest richness, as illustrated in Figure 3A, while the MPL group showed the lowest Shannon index (Figure 3B). Additionally, β-diversity was visualized using a PLSDA plot, revealing four distinct clusters (Figure 3C). ANOSIM analysis confirmed that differences exist among all groups (All *p* < 0.01).

**Figure 4:**
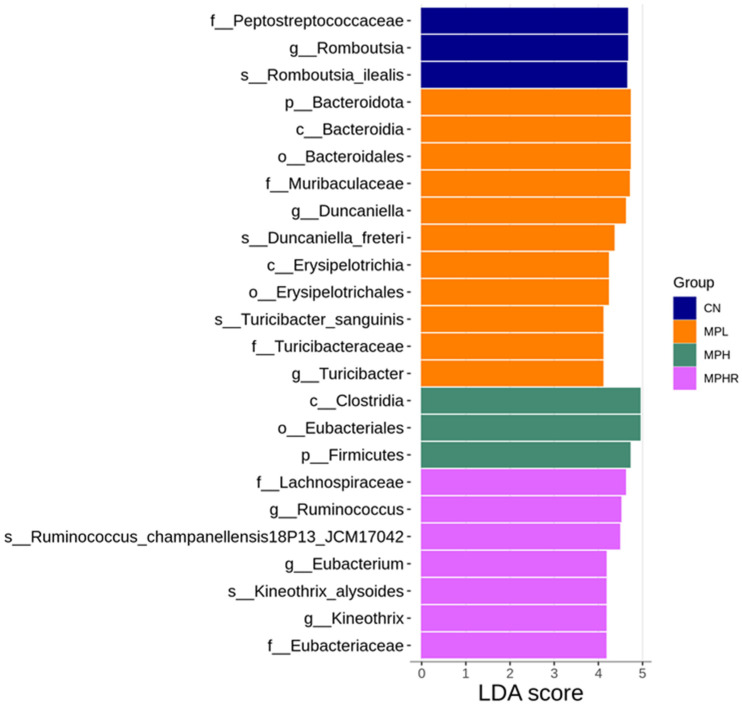
Linear discriminant analysis effect size (LEfSe) analysis indicates the linear discriminant analysis (LDA) score (effect size) showing significant differences in bacterial taxa among the CN (blue), MPL (orange), MPH (green), and MPHR group (pink) (LDA score > 4.0; *p* < 0.05). CN, control rats; MPL, rats received low-dose microplastics; MPH, rats received high-dose microplastics; MPHR, rats received high-dose microplastics and resveratrol.

The LEfSe analysis highlighted the most significant differences in taxa abundance among the four groups, as depicted in Figure 4. Notably, the genera *Duncaniella* and *Turicibacter*, along with their respective family and phylum, were found to be more abundant in the MPL group. Conversely, the genera *Ruminococcus*, *Eubacterium*, and *Kineothrix* were significantly enriched in the MRHR group (LDA > 4, Figure 4).

**Figure 5:**
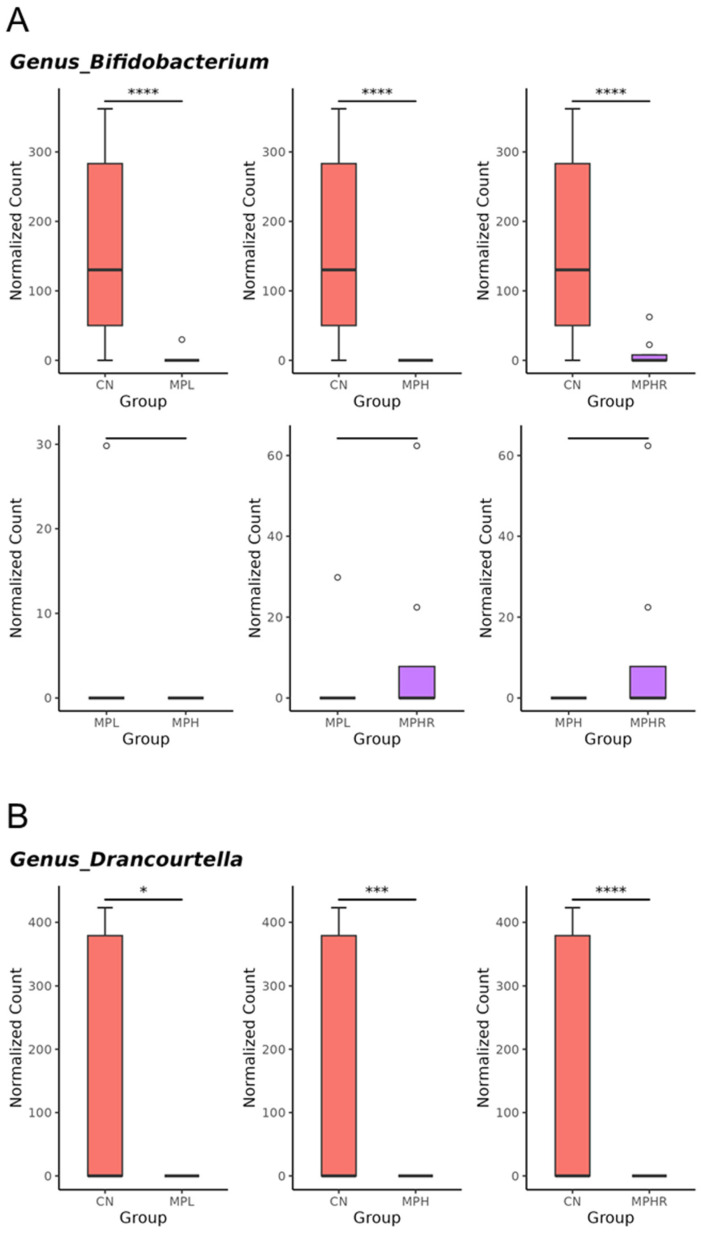

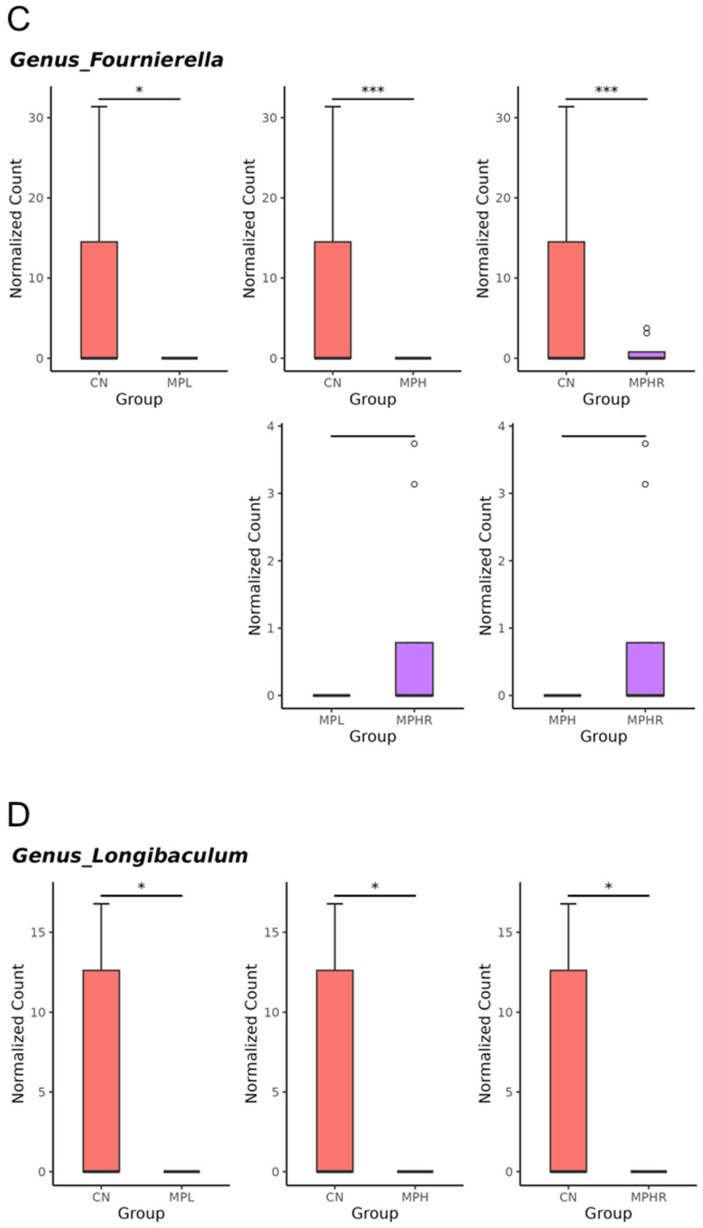

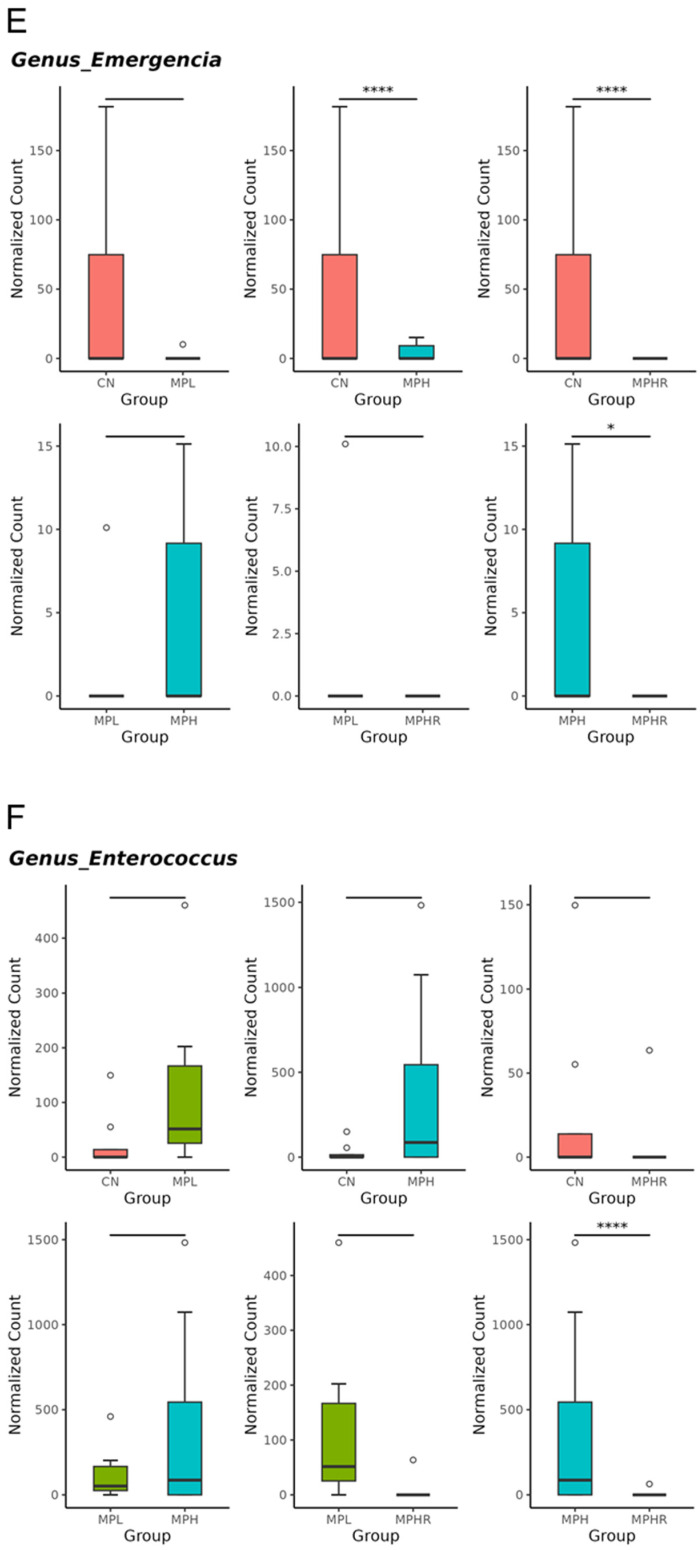

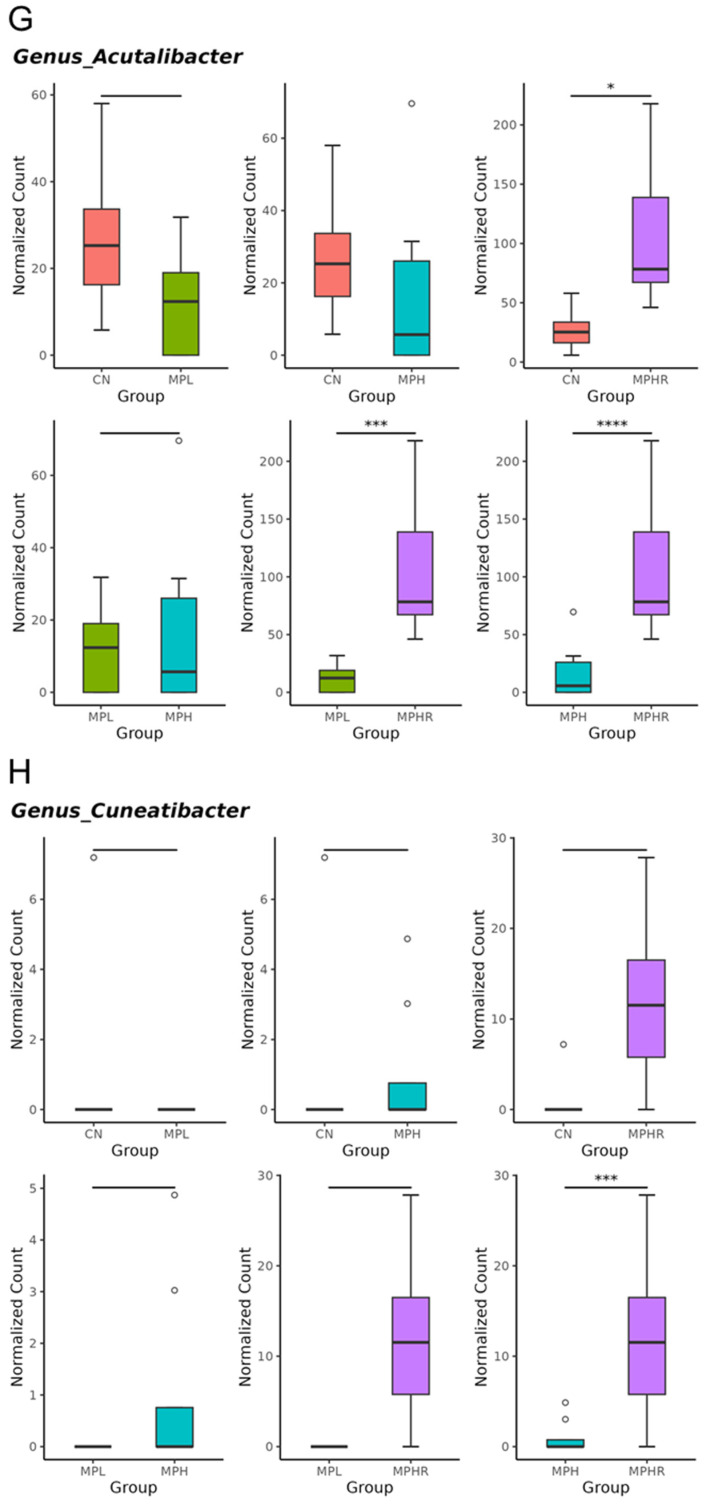
The comparison of genera (**A**) *Bifidobacterium*, (**B**) *Drancourtella*, (**C**) *Fournierella*, (**D**) *Longibaculum*, (**E**) *Emergencia*, (**F**) *Enterococcus*, (**G**) *Acutalibacter*, and (**H**) *Cuneatibacter* among the four groups. The undetectable genus taxa in each group were excluded from the graphs used for group comparison. N = 8/group. * *p* < 0.05; *** *p* < 0.005; **** *p* < 0.001. CN, control rats; MPL, rats received low-dose microplastics; MPH, rats received high-dose microplastics; MPHR, rats received high-dose microplastics and resveratrol.

At the genus level, MP exposure altered gut microbiota composition, resulting in a notable decrease in *Bifidobacterium* (Figure 5A), *Drancourtella* (Figure 5B), *Fournierella* (Figure 5C), and *Longibaculum* (Figure 5D) in both low-dose and high-dose groups. Resveratrol supplementation significantly decreased the abundance of genus *Emergencia* (Figure 5E), *Enterococcus* (Figure 5F), while increased *Acutalibacter* (Figure 5G), and *Cuneatibacter* (Figure 5H) in MPHR rats compared with MPH rats.

### 3.4. Oxidative Stress

**Figure 6:**
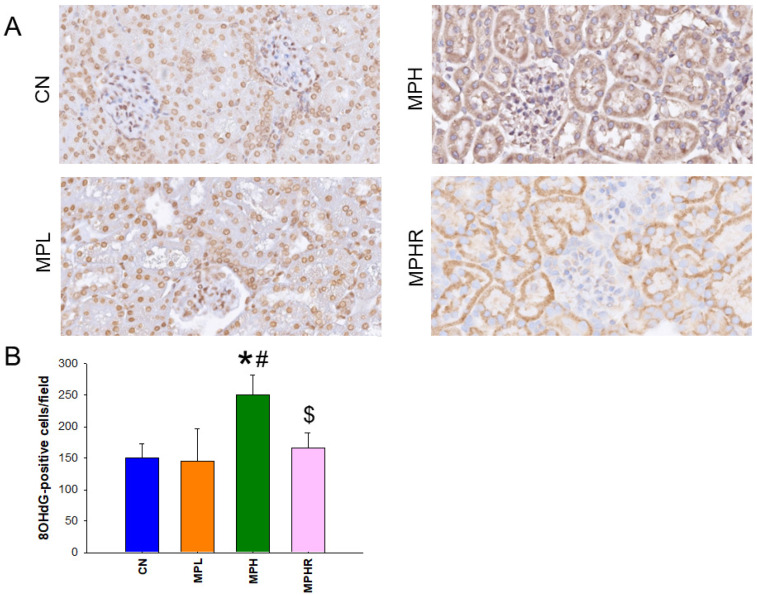
(**A**) Immunohistochemistry for 8-OHdG in the kidneys across four groups revealed that 8-OHdG, a marker of oxidative stress, was localized in the nuclei of tubular cells and glomeruli. (**B**) Quantitative analysis of 8-OHdG-positive cells in the kidney cortex indicated an increase in expression in the MPH group. N = 8/group; * *p* < 0.05 vs. CN, # *p* < 0.05 vs. MPL; $ *p* < 0.05 vs. MPH. CN, control rats; MPL, rats received low-dose microplastics; MPH, rats received high-dose microplastics; MPHR, rats received high-dose microplastics and resveratrol.

We conducted a further analysis of 8OHdG immunoreactivity in the kidneys to evaluate the effects of MP and resveratrol on oxidative stress. As illustrated in Figure 6A, the MPH group exhibited intense staining in renal tubular cells and glomeruli. Additionally, the number of 8OHdG-positive cells significantly increased following high-dose MP exposure compared to the other three groups (Figure 6B).

### 3.5. RAS

**Figure 7:**
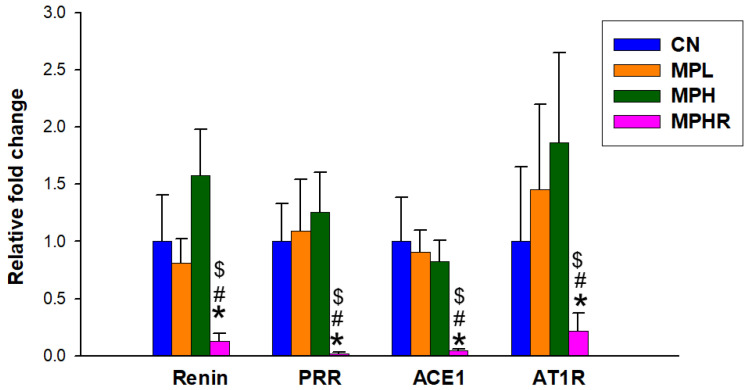
Renal mRNA expression of RAS-related genes, including renin, PRR, ACE1, and AT1R. N = 8/group; * *p* < 0.05 vs. CN, # *p* < 0.05 vs. MPL; $ *p* < 0.05 vs. MPH. CN, control rats; MPL, rats received low-dose microplastics; MPH, rats received high-dose microplastics; MPHR, rats received high-dose microplastics and resveratrol.

We used qPCR to quantify changes in key RAS-related genes in rat kidneys. Figure 7 shows that resveratrol treatment resulted in the lowest renal expression of renin, PRR, ACE1, and AT1R in the MPHR group.

## 4. Discussion

This study presents the first evidence that resveratrol administration mitigates elevated BP and creatinine levels caused by MP exposure in juvenile rats. The beneficial effects of resveratrol are linked to modifications in gut microbiota and its metabolites, reduced oxidative stress, and inhibition of the RAS.

Our key findings include the following observations: (1) MP exposure leads to a dose-dependent increase in BP, with significant kidney function impairment observed only at high doses. (2) Resveratrol supplementation effectively reduces hypertension and restores kidney function compromised by high-dose MP exposure. (3) Hypertension and kidney disease induced by high-dose MP exposure are accompanied by oxidative damage, as evidenced by increased 8-OHdG expression. (4) Low- and high-dose MP exposure, as well as resveratrol treatment, differentially influence gut microbiota composition. (5) Resveratrol’s protective effects against hypertension and kidney damage are accompanied by a decrease in oxidative stress, an increase in plasma acetic acid levels, and reduced renal expression of Olfr78. (6) Resveratrol treatment decreases the abundance of the genera *Emergencia* and *Enterococcus* while increasing levels of *Acutalibacter* and *Cuneatibacter*. (7) Resveratrol administration results in decreased levels of renin, PRR, ACE1, and AT1R.

The association between MP exposure and kidney injury has been documented [7,8,9], though the underlying mechanisms remain inadequately understood. Our findings align with previous studies demonstrating that MP exposure increases BP and creatinine levels in juvenile rats, suggesting that early exposure may contribute to childhood hypertension and kidney disease. Our current study provides the first evidence that resveratrol treatment alleviates hypertension and kidney disease induced by MP exposure in juvenile rats. While previous research has highlighted the antihypertensive and renoprotective effects of resveratrol [10,11,12], our findings specifically demonstrate its protective benefits against hypertension and kidney damage caused by MP exposure.

Prior research suggests that the bioaccumulation and toxic effects of MP in the kidneys may be linked to oxidative stress [8]. Our observations indicate that MP exposure leads to oxidative damage, as evidenced by increased renal expression of 8-OHdG [22]. Notably, only high-dose MP exposure elevates 8-OHdG levels, implying that MP-induced oxidative stress may occur in a dose-dependent manner. Given that MP-induced kidney disease and hypertension are associated with heightened 8-OHdG expression, our study suggests that oxidative stress is a significant pathogenic mechanism. Furthermore, the antioxidant properties of resveratrol are evident, as treatment with resveratrol in juvenile rats mitigates hypertension and kidney dysfunction, coinciding with a reduction in oxidative damage.

Resveratrol treatment also impacts both gut microbiota and their metabolites, as well as the RAS, resulting in lower BP and kidney injury. One of resveratrol’s beneficial effects may be its ability to raise acetic acid concentration while reducing the expression of GPR109A and Olfr78 in the kidneys. SCFAs interact with their receptors to regulate BP [23]. Emerging evidence highlights the potential of SCFAs as postbiotics for managing hypertension and kidney disease [24,25]. Among SCFAs, acetic acid is the most abundant and is known to lower BP through vasodilation [23]. Acetic acid can bind to GPR41, GPR43, and Olfr78, while GPR109A primarily binds to butyric acid [23]. In our study, resveratrol treatment increased acetic acid levels, which were reduced by MP exposure. Additionally, resveratrol treatment decreased the renal expression of Olfr78, which is known to influence renin secretion and increase BP [26]. These findings are consistent with previous research suggesting that acetic acid supplementation can help prevent hypertension by restoring dysbiotic gut microbiota involved in BP regulation [27,28,29]. Therefore, the overall effects of resveratrol on acetate production and its modulation of Olfr78 may contribute to its protective benefits against hypertension in this model.

So far, data on the impact of MPs on gut microbiota remain limited [30]. Our findings indicate that MP exposure induces hypertension and kidney disease, which are associated with a depletion of SCFA-producing bacteria such as *Bifidobacterium*, *Drancourtella*, and *Fournierella* [31,32,33]. Additionally, the genus *Turicibacter*, along with its respective family and phylum, was found to be more enriched in the MPL group. This aligns with previous research demonstrating that MPs can affect soil microbial communities, leading to an increase in *Turicibacter* abundance [34]. However, the roles of these MP-induced taxonomic changes in the development of hypertension and kidney disease have not yet been thoroughly investigated.

Resveratrol is also protective due to its effects on gut microbiota. We observed that resveratrol increased the abundance of the known probiotics *Ruminococcus* and *Eubacterium* [35]. Additionally, our data indicated a reduction in the proportion of the genera *Emergencia* and *Enterococcus*. *Emergencia timonensis*, a key bacterium that converts carnitine to trimethylamine N-oxide (TMAO) [36], is known to exacerbate hypertension and kidney damage [37]. Similarly, *Enterococcus* spp. are associated with hypertension and renal injury [38]. Therefore, further investigation is needed to determine whether resveratrol protection against hypertension and kidney disease is attributed to the reduction in these specific microbes. Conversely, resveratrol treatment increased the abundance of the genera *Acutalibacter* and *Cuneatibacter*. *Acutalibacter* has been identified as a microbial marker for colorectal cancer [39], while *Cuneatibacter* is known for its SCFA production. However, the roles of these genera in the protective actions of resveratrol against hypertension and kidney disease remain unclear.

Another possible advantage of resveratrol is its capacity to block the classical RAS. The RAS cascade initiates when circulating renin interacts with the PRR, resulting in the local production of angiotensin II (Ang II). This peptide is a key factor in the development of hypertension and kidney disease, primarily through the classical RAS pathway, which involves ACE1, Ang II, and AT1R [40]. We observed that resveratrol treatment inhibited the expression of renin, PRR, ACE1, and AT1R, even though the RAS did not appear to be upregulated by MP exposure.

We acknowledge several limitations in our study. Firstly, we did not investigate female rats, leaving it unclear whether sex differences exist in the effects of MP exposure and the bioactivity of resveratrol, which warrants further research. Another limitation is that our study focused solely on SCFAs. It is important to note that a comprehensive analysis of all microbial metabolites in a single study has not yet been reported. While our research provides evidence for the protective effects of resveratrol on SCFAs and their receptors, its impact on other microbial metabolites requires further investigation. Although our results indicate that MPs caused dose-dependent increases in oxidative damage and hypertension, the study has limitations, including a lack of a thorough examination of oxidative stress mechanisms. Further research is needed to clarify which aspects of radical signaling and the antioxidant defense system are key targets, potentially offering specific strategies for managing MP exposure-related hypertension and kidney disease. Fourthly, evidence suggests that in some hypertension models, elevated BP detected by the tail-cuff method may not be observed in telemetrically instrumented animals [41]. These results indicate that part of the increase in BP may be due to a heightened stress response.

While this work provides critical insights into the potential risks of MP exposure, translating these animal findings to humans requires careful consideration of species differences, exposure routes, and environmental relevance. Our study represents an important step in understanding the effects of MPs, but further research—including human studies and more advanced models—is needed to fully assess their impact on kidney health, particularly in children.

Lastly, although our results highlight the beneficial effects of resveratrol on hypertension and kidney disease induced by MP exposure in this specific model, it is important to recognize that these results may not directly translate to human kidney function or disease. The physiological differences between juvenile rats and humans, as well as variations in metabolic pathways and responses to treatment, can impact the applicability of animal study results to human health. Hence, additional research is needed in various animal models of MP exposure to elucidate potential mechanisms, followed by studies involving human participants, before resveratrol can be considered for clinical application.

Although many in vitro and animal studies suggest that resveratrol may offer health benefits, human clinical trials have produced mixed results regarding its protective effects against diseases and their sequelae [42,43,44]. Most human studies have failed to demonstrate the same level of effectiveness seen in preclinical models. The reasons for these discrepancies are multifactorial, with factors such as differences in patient characteristics, resveratrol dosage, and the duration of supplementation being among the key variables proposed to explain the conflicting findings [44]. These variations highlight the need for more rigorous, large-scale studies to better understand both the potential and the limitations of resveratrol in human health.

Furthermore, many of resveratrol’s dose-dependent responses, both in vitro and in vivo, appear to follow a hormetic dose-response pattern, where low doses induce beneficial effects, while higher doses may lead to adverse outcomes [45]. While a 50 mg/L dose used in the current study is commonly applied in rat studies to assess cardiovascular and renoprotective benefits [46], the optimal human dose—one that provides therapeutic effects without causing toxicity—remains unclear. Determining this ideal dosage is a critical area of ongoing research [47].

## 5. Conclusions

This study not only reinforces the finding that early MP exposure leads to hypertension and kidney disease in juvenile rats but also demonstrates that resveratrol treatment offers protective effects. Although the mechanisms underlying MP-induced kidney damage and hypertension are not yet fully understood, our findings suggest that the beneficial effects of resveratrol are connected to changes in gut microbiota and SCFA signaling, reduction of oxidative stress, and inhibition of the RAS. Thus, resveratrol supplementation may be a viable approach for supporting optimal kidney health in the context of plastic pollution among children.

## Figures and Tables

**Table 1:** Primer sequences for qPCR analysis.

Gene	Sense	Antisense
GPR41	TCTTCACCACCGTCTATCTCAC	CACAAGTCCTGCCACCCTC
GPR43	CTGCCTGGGATCGTCTGTG	CATACCCTCGGCCTTCTGG
GPR109A	CGGTGGTCTACTATTTCTCC	CCCCTGGAATACTTCTGATT
Olfr78	GAGGAAGCTCACTTTTGGTTTGG	CAGCTTCAATGTCCTTGTCACAG
Renin	AACATTACCAGGGCAACTTTCACT	ACCCCCTTCATGGTGATCTG
PRR	GAGGCAGTGACCCTCAACAT	CCCTCCTCACACAACAAGGT
ACE1	CACCGGCAAGGTCTGCTT	CTTGGCATAGTTTCGTGAGGAA
AT1R	GCTGGGCAACGAGTTTGTCT	CAGTCCTTCAGCTGGATCTTCA
R18S	GCCGCGGTAATTCCAGCTCCA	CCCGCCCGCTCCCAAGATC

**Table 2:** Plasma SCFA levels at age 9 weeks.

Group	CN	MPL	MPH	MPHR
Acetic acid (μM)	1181 ± 66	951 ± 52 *	1045 ± 70	1316 ± 66 #$
Propionic acid (μM)	5.6 ± 0.4	5.6 ± 0.5	5.7 ± 0.3	6.3 ± 0.4
Isobutyric acid (μM)	3.2 ± 0.2	3.3 ± 0.1	3.6 ± 0.3	3 ± 0.1
Butyric acid (μM)	16.1 ± 1.7	16.6 ± 1.5	15.1 ± 1.4	15.5 ± 1
Isovaleric acid (μM)	5.7 ± 0.4	5.1 ± 0.7	4.4 ± 0.3 *	5 ± 0.3
Valeric acid (μM)	7.9 ± 0.9	9 ± 0.5	7.2 ± 0.8	7.9 ± 0.8

CN, control rats; MPL, rats received low-dose microplastics; MPH, rats received high-dose microplastics; MPHR, rats received high-dose microplastics and resveratrol. N = 8/group; * *p* < 0.05 vs. CN, # *p* < 0.05 vs. MPL; $ *p* < 0.05 vs. MPH.

## Data Availability

Data are contained within the article.
